# Social inclusion revisited: sheltered living institutions for people with intellectual disabilities as communities of difference

**DOI:** 10.1007/s11019-022-10135-7

**Published:** 2022-12-30

**Authors:** Femmianne Bredewold, Simon van der Weele

**Affiliations:** Chair Group Citizenship and Humanisation of the Public Sector, University of Humanistic Studies, Utrecht, The Netherlands

**Keywords:** Social inclusion, Intellectual disability, (De)-institutionalisation, Difference, Empirical ethics

## Abstract

The dominant idea in debates on social inclusion of people with intellectual disabilities is that social inclusion requires recognition of their ‘sameness’. As a result, most care providers try to enable people with intellectual disabilities to live and participate in ‘normal’ society, ‘in the community’. In this paper, we draw on (Pols, Medicine Health Care and Philosophy 18:81–90, 2015) empirical ethics of care approach to give an in-depth picture of places that have a radically different take on what social inclusion for people with intellectual disabilities looks like: places known as ‘sheltered living institutions’. We argue these places can be seen as ‘communities of difference’ catered to the specific needs and capacities of the residents. We then contend that these communities raise questions about what a good life for people with intellectual disabilities looks like and where and how it ought to be realised; questions not posed very often, as they get muzzled by the dominant rhetoric of normalisation and the emphasis on sameness.

## Introduction

For decades, parents, relatives, social professionals, policymakers, and academics have been debating what constitutes ‘a good life’ for people with intellectual disabilities.^1^ Looking back to history, we can conclude that responses to this question are related to how people with disabilities are perceived, with these perceptions being socially and culturally constructed (Arneil 2009). In Western societies, the discussion about what constitutes a good life has been centred—at least since the 1960s—around the goal of ensuring ‘social inclusion’ (Culham and Nind 2003; Stiker 1999).

We can roughly distinguish between two opposing ideals of how a good life for people with intellectual disabilities can be facilitated by social inclusion (Arneil 2009; Bos and Kal 2016; Bredewold 2021; Meininger 2010; Silvers 1995; Stiker 1999). On the one hand, we find the argument that social inclusion requires an emphasis on the *sameness* of people with and without intellectual disabilities; on the other, there is the argument that social inclusion requires making room for *difference*. This ‘sameness-difference debate’ has emerged similarly in many other academic fields concerned with minority groups, and Iris Marion Young summarises it as follows: ‘A politics of sameness assumes that equal social status for all persons requires treating everyone to the same standards, principles and rules. A politics of difference argues that equality can be gained by requiring different treatment for disadvantaged and marginalised groups’ (1990, p. 158). Young and others have recognized that the opposites of sameness and difference are ideal-types, which are frequently negotiated or compromised in practice (Young 1990; Arneil 2009; Bredewold 2021). Following them, we employ sameness and difference as ideal–typical frames to outline the historical debate on how social inclusion for people with intellectual disabilities ought to be realized, recognizing that things are often more muddled in practice.

Today, the dominant ‘good life ideal’ guiding policy and practice in Western societies tends towards the ‘sameness’ view of social inclusion. This emphasis on sameness emerged in the 1960s and can be seen as a marked reaction against the practice of institutionalisation, in which the label of intellectual disability was used to categorise people as ‘abnormal’, ‘deviant’, and ‘not like us’ (Goffman 1963). The critique of this dehumanisation of people in institutions gave rise to the deinstitutionalisation and normalisation movements, in which ‘sameness’ became the basis for social inclusion (Nirje 1985; Wolfensberger 1972).^2^ This movement advocates for people with intellectual disabilities to be treated as human beings who deserve to be empowered to fully participate in society and fulfil valued roles within it (Bigby et al. 2015; Nirje 1985; Taylor and Bogdan 1989). Importantly, this also means giving people with intellectual disabilities the legal and human rights shared by all other citizens (Culham and Nind 2003). In most Western countries, from the 1960s onward, institutions have been dismantled to encourage people with disabilities to participate in mainstream society and live ‘normal’ lives ‘in the community’, just like other people (Bredewold 2021; Bredewold, Hermus and Trappenburg, 2018; Van der Weele et al. 2021).

However, in the Netherlands (and in many other countries; Mansell 2006; Wiesel and Bigby 2015), the institutions remained—despite widespread international acceptance of deinstitutionalisation policies and practice. In the 1990s, the number of Dutch people with intellectual disabilities living in institutions actually increased (Beltman 2001, p. 210). This was the result of lobbying by institutions, resistance groups formed by concerned parents, a lack of financial incentives, and the conflicting interests of various government departments (Beltman 2001, p. 209–211). As a result, by 2015, approximately 20,000 of the 142,000 Dutch people with intellectual disabilities were still living in institutions (Mans 2016).

Notably, these remaining institutions do not look anything like those of the 1960s and earlier. Although they are still typically situated in predominantly rural areas, and in some ways secluded from ‘mainstream’ society, the institutions have modernized to meet contemporary Western standards for quality of life. Residents live in the privacy of their own space (usually in a group home setting), they have jobs (typically on-site and occasionally elsewhere), and they have access to a variety of pastime clubs and activities. Relatives are free to visit at all times, the residents are free to leave the grounds, and staff are trained to the same standards as support workers operating ‘in the community’. Distinctive amongst institutions, however, is that all facilities for daily living are present within the grounds: in addition to the group homes and workplaces, most sites also include a healthcare centre, a general store or supermarket, a place of worship, and various places for pastime activities, such as a pool, a theatre, riding stables, and a petting zoo. It is for this reason that the institutional grounds are sometimes referred to in Dutch as *beschermende leefgemeenschappen* or ‘sheltered living communities’.

Unsurprisingly, these remaining institutions are the subject of some controversy, both in the Netherlands and internationally (Mansell 2006; Wiesel and Bigby 2015). As life in these places eschews the practice of organizing life ‘in the community’, they appear anathema to the principle of sameness that currently dominates debates on configuring a ‘good life’ for people with intellectual disabilities. This ethical tension stood at the basis of a recent multi-year ethnographic research project in which we investigated what daily life for people living in these institutions looks like and how managers, professionals, and relatives envision a ‘good life’ for them. Our project was driven by the question of how these institutions fit into the debate about the social inclusion of people with intellectual disabilities and the related discussion about sameness and difference.

In this article, we focus on three of the six sheltered living institutions that were part of our study and which confronted us with a radically different take on what a good life for people with intellectual disabilities might be. These three sheltered living institutions assume that the achievement of equality requires ‘difference’ as the leading concept, and daily life in the institutions is organized around this principle. We claim that, in this way, the institutions are effectively *heterotopias*, or ‘places of difference’ (Foucault 1967, 1970, 1986). Heterotopias are literally ‘other spaces’ (derived from the Greek *heteros* [‘another’] and *topos* [‘place’]), which can be understood as ‘counter-sites’ in which the order of things is challenged (Johnson 2006, 2013). Such spaces can thus also function as a critical mirror for prevailing and dominant practices. This is how we will approach them in what follows. If, as our informants claim, institutions must be regarded as heterotopias, what does the critical mirror they uphold show us?

We explore this question below, by looking into the daily care practices in these institutions and the goods they bring forth through the lens of the ‘empirical ethics of care’ (Pols 2015). Our aim is to examine how and to what extent these practices might critically interrogate dominant ideas about ‘normalcy’ and the good life for people with intellectual disabilities. Moreover, our research raises questions about how we understand and deal with ‘difference’ in Western societies. However, it is emphatically not our goal to pass judgement on the desirability of sheltered living institutions in the landscape of intellectual disability care. In line with the ‘empirical ethics of care’ approach, we do not aim to formulate prescriptive conclusions (Pols 2015, p. 82). Rather, we explore the normative repercussions of organizing care through alternative notions of the good life for people with intellectual disabilities to shed new light on the debates on sameness-difference and social inclusion.

## Social inclusion: a struggle between sameness and difference

Owing to the influence of the normalisation movement, the discussion about a good life for people with intellectual disabilities is largely dominated by the idea that it is important to emphasise sameness (Bredewold 2021). Under the broader concept of ‘social inclusion’—which is mainly understood as ‘participation in society and interpersonal relationships with other people in the community’ (Simplican et al. 2015, p. 19)—this emphasis results in stimulating people with intellectual disabilities to live and work ‘in society’, participate ‘in the community’, and enlarge their social networks to include people without intellectual disabilities.

However, research has shown that it is difficult to achieve social inclusion on the basis of normalisation. People with intellectual disabilities are present in the community, but their community participation is limited. Their contacts are usually limited to family members, other people with intellectual disabilities, and support staff (Bigby 2008; Forrestor-Jones et al. 2006; Bredewold et al. 2018). Moreover, accounts of social inclusion based on normalisation have been tainted with controversy, as such accounts fail to consider what to do when people cannot live up to the demands of ‘normalcy’. Furthermore, it has been questioned why normative ideals should be set solely by people without intellectual disabilities. There are also concerns that normalisation implies changing and ‘normalising’ individuals’ differences, obliging them to conform in exchange for acceptance (Culham and Nind 2003; Hall 2004; Meininger 2010; Oliver 1993; Simplican et al. 2015; Stiker 1999).

Much of the criticism of normalisation is associated with the emergence of the social model of disability, which criticizes the medical model in which people are cast as individually lacking and instead sees society as disabling (Culham and Nind 2003, p. 68; Oliver 1993). Fuelled by the criticism of the social model, questions arose such as, does normalisation mean that people with an intellectual disability should be ‘normalised’ and indeed forced to act ‘normal’? Nirje and Wolfensberger, founders of the normalisation movement, were sensitive to this criticism and sought to disassociate their models from it. Nirje (1985, p. 69–70) states that ‘normalisation’ does not mean that people should be ‘normalised’, but rather refers to the acceptance of people with disabilities within ‘normal’ society, while Wolfensberger abandoned the term ‘normalisation’ and adopted ‘social role valorisation’ in its place (Culham and Nind 2003; Wolfensberger 1983).

Although the founders of the normalisation movement were sensitive to this criticism about the denial of difference, it remains relevant. Henri-Jacques Stiker (1999) argues that modern society’s wish is ‘to make people with intellectual disabilities identical, without making them equal’ (1999, p. 150–151). According to Stiker, in society, ‘the measures and actions on behalf of “impaired citizens” tend to efface their difference but not establish them on the same level economically and socially’; and hence, ‘we are shaping a society in which there is no plan for equality but one of identicalness’ (p. 150–151). More recently, Meininger (2010) has stated that ‘a re-conceptualisation of social integration is very much needed’ (p. 192), inviting the inclusion of the ideals of people with intellectual disabilities. These scholars are not alone in calling for a discourse in which societies make difference the norm. We find increasing numbers of disability scholars concluding that, to ensure the equality of people with intellectual disabilities, it is important to recognise how they differ and how their difference can be accommodated (e.g. Arneil 2009; Reerink et al. 2017).

However, even if scholars increasingly embrace in theoretical debates the idea of difference as a way of reaching equality, the principle of sameness dominates the agenda of practice-oriented social-scientific research. Places that go against ‘normalising’ care principles and do not aim to include people with intellectual disabilities in ‘mainstream’ society are virtually unstudied, with only a few exceptions. Hall (2004, 2010) investigated places in society deemed ‘intellectual disability exclusive’ (Hall 2004, p. 54) and states that these kinds of places are important for people with disabilities. He found that a significant number of people with intellectual disabilities lacked the desire or ability to reach the standards set by social inclusionary policy. Therefore, Hall proposes the creation of ‘spaces of belonging’ (Hall 2004, 2010): ‘spaces within which people with intellectual disabilities can experience belonging, without being exposed to the rigors associated with “normal” social inclusionary positions’ (Hall 2010, p. 56). He emphasises that these safe spaces should not be understood as inferior substitutes for participation in mainstream society, but rather as alternative spaces that reflect different identities.

McKearney (2017) conducted fieldwork in the Christian L’Arche communities in the United Kingdom, in which people with intellectual disabilities live together in residential group homes. In his view, these communities—in which dependence on others is not an obstacle but rather a vehicle for recognition—form a counterpoint to more common care-providing practices that aim to stimulate people with intellectual disabilities to master independence. He argues that we need alternative care models such as L’Arche, as ‘the normalisation movement aims to cultivate in people with learning disabilities an autonomy they may never develop, in order to integrate them into urban settings over which they have no control, and that can be hostile to their ambitions for friendship and participation’ (McKearney 2017, p. 274). According to McKearney, in alternative forms of care such as L’Arche, people with disabilities can gain a positive self-definition, as their dependent relationships with peers can show them that they are of value as people and are valuable for one another.

Thus, scholars such as Hall and McKearney argue that places operationalizing the principle of ‘difference’ over ‘sameness’ enable people with intellectual disabilities to gain a positive self-definition—hich, they posit, is necessary to reach equality and social inclusion on the terms set by the people with intellectual disabilities themselves. They also show—and we strongly agree—that the principles of ‘sameness’ and ‘difference’ are not reducible to an essential dichotomy between ‘living in the community’ and ‘living in institutions’, in which the former figures as an elaboration of ‘sameness’ and the latter as an elaboration of ‘difference’. The logic of ‘difference’ can and does enter community care.

As pointed out above, few researchers have studied places committing wholly to the principle of ‘difference’, as these are overshadowed by the dominant rhetoric of normalisation and the emphasis on sameness. Following Hall and McKearney, in this paper, we take a closer look at alternative forms of care that appear to accommodate difference. However, in our research, we turn away from community care to examine specific forms of institutional care.

## Methods

The ethnographic fieldwork we discuss in this article took place in 2019 at six sheltered living institutions in the Netherlands, each of which was run by a different care organization. Our goal was to examine the daily life in these institutions and consider how it was shaped by ideals about what a ‘good life’ for people with intellectual disabilities should entail. In this article, we focus on three of the sheltered living institutions that were part of our study. These three organisations had very specific organisational identities: two were informed by anthroposophical philosophy and one by a self-formulated philosophy rooted in the thought of various well-known thinkers, such as Emmanuel Lévinas and Dorothee Sölle. In addition, while the other three organizations we studied also provided community care, the three we discuss here exclusively managed institutional grounds. We choose to home in on these three organizations because they confronted us with a radically different take on what a good life for people with intellectual disabilities might be, seemingly committing wholly to the principle of ‘difference’. In this, they differed from the three other organizations we examined, which found themselves involved in a balancing act between ‘sameness’ and ‘difference’ (Bredewold and van der Weele forthcoming). Because they are so different, they hold up a mirror and reveal a new perspective on the debates on sameness-difference and social inclusion.

All three institutions we studied were home to a highly diverse group of people with intellectual disabilities, with various levels of support needs, ranging from mild to profound disability and everything in between. In general, residents of sheltered living institutions require a so-called ‘Wlz-indication’, which makes use of the Dutch Long-Term Care Act [*Wet Langdurige Zorg*]. In practice, this meant that all the residents in the institutions we visited were adults who needed around-the-clock care and support. In the Netherlands, people with intellectual disabilities and their relatives have the right to choose a care provider according to their own preference. The three institutions discussed here all accepted enrolments of prospective residents on their websites. Due to their popularity, these institutions tended to have long waiting lists.

We spent one week at each institution, during which time we followed a total of nine residents, using a method known as shadowing (Van der Weele and Bredewold 2021; McDonald 2005; Quinlan 2008). This is a form of participant observation in which the researcher follows a single person over an extended period of time to get a sense of their daily life and relationships. By spending these weeks shadowing residents, we were able to participate in the everyday ebb and flow of events and to grasp how these events affected and were experienced by the residents we followed. Prior to shadowing, we obtained written ethical approval.

In addition, we interviewed a support worker and relative of each person we shadowed. In these interviews, we asked our informants about their views of life in the institution and about their vision on what a ‘good life’ for the resident would look like. We also interviewed the director(s) of each participating care organization to ask them about their perceptions of life in the institution grounds and about the ideals that informed the organization’s approach to support services. Prior to interviewing, respondents signed a document declaring that they had been informed about the aims of the research, that they gave permission to take part, and could withdraw this permission at any time in the future. When prospective participants did not have the legal status to consent, permission was obtained from a guardian. All observations were anonymised. We use pseudonyms throughout this article.

Since our interest here lies in the ideals informing the organization of the institutions we examined, our focus in this paper is on the perspective of support workers, relatives, and managers—who together tend to be largely responsible for the institution’s organization. Our aim, then, is not to pass judgement on the institutions or to assess how people with intellectual disabilities feel about them. Rather, it is to uncover the tacit moral logic that shapes their everyday life.

To make sense of our observations, we adopted an analytical approach known as ‘empirical ethics of care’ (Ceci et al. 2017; Pols 2015). This approach has as its central premise that ‘[c]are practices have a normative orientation towards some kind of good’ (Ceci et al. 2017, p. 57) and that care is geared towards ‘putting something good into practice’ (Pols 2015, p. 83). The goal of the empirical ethics of care is to study what these goods are and how they unfold in specific care practices. This is not to say that the outcomes of care are necessarily ‘good’; it is merely to suggest that care practices are indicative of the values, strivings, and preferences of those who are putting care into practice. Studying the goods enfolded in care practices can thus instruct the researcher on the ideals and values that undergird these practices. Notably, these practices include not only the doings and goings of the care workers, but also the material dimensions of care, such as buildings and objects. The empirical ethics of care approach thus homes in on everyday moral life, regarding human beings as evaluative beings who look to bring something good in practice. In this way, the approach broadly aligns with recent work in the field of moral anthropology (Lambek et al. 2015; Mattingly and Throop 2018).

By adopting an empirical ethics of care approach, we were able to observe daily life at the institution as expressive of a particular set of ideals and values and of a vision of what a ‘good life’ for people with intellectual disabilities should entail. By comparing these observations with the ideas that our informants articulated in the interviews about this ‘good life’, we could study both the ideals that supposedly give rise to life at the institution and how (and whether) these are actually translated into care practices.

Importantly, the empirical ethics of care approach does not understand normativity as a judgement made by ethicists or researchers after a careful weighing of principles. It does not want to ‘separate a world out there and the ethics added to it’ (Pols 2015, p. 83), nor posit the researcher as the final moral authority on good care practices (Van der Weele 2022). Instead, this approach aims to outline and describe different ideas of what constitutes good care in practices so that comparisons can be made, not just by researchers but also by those involved in these care practices. This is also what we aim for in this article. By describing the ‘goods’ operant in these sheltered living institutions, we seek to pose questions about what a good life for people with intellectual disability might look like. Thus, our analysis will not pass judgement on the desirability of sheltered living institutions as such, but rather home in on their unusual approach to care to critically interrogate dominant ideas about ‘normalcy’ and the good life for people with intellectual disabilities.

## Findings

### Life in a ‘mini-society’: sheltered living institutions as heterotopias

As we mentioned in the introduction, the principal ideal underpinning visions of the ‘good life’ for people with intellectual disability today is that of sameness. This principle dominates policy, practice, and popular discourse. What stood out from the interviews with our informants, then, was how emphatically they distinguished sheltered living institutions from the rest of Dutch society as places of *difference*. They repeatedly stressed that these places differed from wider society and formed worlds of their own. They emphasised that the difference of people with a disability is accommodated in these places. This was reflected in their language. Managers, support workers, and relatives alike referred to the institution using phrases such as ‘a small society’, ‘a mini-society’, ‘their own society’, ‘a small village’, ‘a society within society’, and so on. This place was contrasted with what they called ‘external society’, ‘the bad outside world’, ‘the normal world’, ‘the big world’, ‘the big society’, and so on:

Let’s say everything that’s outside of this terrain is society, and this is just a kind of… little society, right? (P13, professional).

We actually have a small society here that offers all the facets of the big society, only it’s adapted to the needs of people with intellectual disabilities. (B1, manager)

In conversations about living in the institution, the world of the sheltered living institution was contrasted with ‘normal’ society. This distinction was not merely semantic; it had clear practical ramifications. Managers, support workers, and relatives frequently emphasized that sheltered living institutions were intentionally arranged in ways that differed from how life is organised in Dutch neighbourhoods and in Dutch society at large. In the ‘normal world’, life is adapted to the standards of the ‘outside’. In the institution, on the other hand, life is attuned to the capabilities and needs of residents themselves. They thus framed the institution as a place that respected the *difference* of people with intellectual disabilities, and they saw a ‘good life’ as one in which these differences were accommodated.

Explaining the need for places of difference, our informants emphasized that ‘the normal world’ or ‘external society’ was simply too hostile for people with intellectual disabilities. They explained that ‘normal’ Dutch society has various characteristics that mean it does not meet the needs of their residents. They referred to Dutch society as individualistic, unwelcoming, unfriendly, busy, and confusing. They even felt that living in the community would be unsafe.

If you look at what inclusion would mean for some people in a society much too crowded, too complicated, unsafe, and sometimes very unfriendly… It’s not because people don’t want to be nice to people with intellectual disabilities, but if you don’t know them, you just don’t understand… Sure, you can have a cup of coffee once or greet each other in the store, but even that is very difficult in our society. There’s only one regular cash register left at the supermarket, all others are self-scan. So that’s where our residents go. Well, is that inclusion? (V6, relative)

For this reason, informants were keen to stress that social inclusion in the community is not realistic, or even an outright policy failure. Integration, they argued, is impossible if the ‘outside world’ has no interest in accommodating difference. They recalled numerous incidents of people with disabilities encountering abuse, harassment, and exploitation while living in the community. They also gave examples of people who had lived in ‘normal’ neighbourhoods and then chosen to return to the sheltered living institutions because they had found ‘normal’ society too unwelcoming.

It often starts very nicely. People start by moving around well in that society, but the result—at least, what we hear and see a lot—is that they become stagnant, lonely, have no connection with the neighbourhood, with the people around them, and life becomes too complicated. (B1, manager)

In short, the managers, support staff, and relatives contrasted sheltered living institutions with what they called ‘normal society’ and argued for the need to create a place that made room for what they believed people with intellectual disabilities needed for a ‘good life’.

By contrasting sheltered living institutions so sharply with ‘normal society’, our informants essentially designated these spaces as standing outside the normal order of things. In this, their perception of an institution resembles what Michel Foucault (1967, 1970, 1986) calls a *heterotopia*. In ‘Different Spaces’ (1986) Foucault introduces the concept of heterotopia as follows:

In every culture there are ‘real places’—places that do exist and that are formed in the very founding of society—which are something like counter-sites, a kind of effectively enacted utopia in which the real sites, all the other real sites that can be found within the culture, are simultaneously represented, contested, and inverted. Places of this kind are outside of all places, even though it may be possible to indicate their location in reality. Because these places are absolutely different from all the sites that they reflect and speak about, I shall call them, by way of contrast to utopias, heterotopias (p. 178).

Heterotopias are thus defined as places of ‘difference’. And as difference never stands alone, this raises the question of what they are different in relation to (Johnson 2016, p. 3). As the concept is only briefly sketched by Foucault (Johnson 2006, 2016), this leaves an opportunity to configure this relationship in various ways. Some authors argue that heterotopias are sites of resistance to the dominant culture, while others regard them as reflecting ordinary life, modernity, late capitalism, a perfectly ordered society, or the opposite of society (Johnson 2006, p. 81–83). At any rate, most agree that there is a persistent association with spaces of resistance and transgression (Hetherington 1997; Johnson 2006, p. 81). Foucault talks of a space that somehow ‘challenges or contests the space we live in’ (Foucault 1986, p. 178). These places can thus be understood as ‘counter-sites’ in which the order of things is challenged (Johnson 2006, 2013, 2016): they ‘reflect’, ‘mirror’, and ‘contest’ (Foucault 1986, p. 178). Johnson states that ‘heterotopias are not separate from dominant structures and ideology’, but ‘go against the grain’ and ‘offer lines of flight’ (Johnson 2006, p. 87). This marks heterotopias as spaces that can challenge the established order of things (Foucault 1970).

Our respondents clearly regarded the sheltered living institution as one such ‘counter-site’: they repeatedly emphasized the ways in which these spaces were distinct from what they referred to as ‘normal society’ and argued that they provided a critical alternative to what ‘normal society’ had to offer.

Two points are of note here. First, in organizing care for people with intellectual disabilities in an institutional setting, our respondents expressed allegiance to the principle of *difference* rather than that of *sameness*: they perceived sheltered living institutions as spaces attuned to the difference of people with intellectual disabilities and forming a clear alternative to what they called ‘normal society’, in which the principle of sameness was dominant. In their view, they had considered the relevant differences of people with intellectual disabilities and, inspired by this, adapted the living environment and care practices to the needs and capacities of the residents. The interviewees occasionally referred to the normalisation principle—inspired by the idea of emphasising sameness to gain equality—as unsuited to the needs of the people concerned. They questioned—in line with academics such as Stiker (1999) and Meininger (2010)—why normative ideals seemed to be set solely by people without intellectual disabilities and what to do when people were not able to live up to these norms:

I think, yes, living here [*at the sheltered living institution*] is much better than living in a village where you need to normalise, while people with intellectual disabilities are not normal and cannot normalise; they just cannot. (P10, professional)

In this sense, the institution was seen by most of our respondents as a place that diverged from the principle of sameness that dominates thinking in ‘regular society’ about the provision of support for people with intellectual disabilities.

Second, although the managers, support workers, and relatives seemed to be driven by a belief that difference must be emphasized and accommodated, this did not mean that the values and goods they embraced radically contradicted those that are embraced when emphasising sameness. Our informants made it clear that, in their institutions, the stimulation of social contacts, participation, and encouragement to contribute were valued in the same way that they were elsewhere. An important difference, however, is that our informants believed that these values and goods had to be defined on the terms of the people with disabilities themselves. This makes the institutional space distinct from ‘ordinary’ society. In other words, what differentiated the institution from ‘regular society’ in the eyes of our informants was not necessarily the ‘goods’ of good care as such, but rather how these goods were put into practice to adapt to the needs of people with disabilities. This raises the question of how (if at all) the values of our informants were translated into the practice of everyday life. What—if anything—makes the sheltered living institution a heterotopia in practice? To explore these questions, we turn to our observations in the field.

#### Crafting the heterotopia in everyday life

As we embarked on our fieldwork, we soon found that life in the institutions is profoundly different from life ‘in the community’. But, as was said earlier, this is not because the pursued ‘goods’ as such were altogether different from support work ‘in the community’. For this reason, a focus on goods would not adequately depict the nature of life in the sheltered living institutions. Rather, these goods must be seen in light of the overarching aim that informed almost everything that happened in the places we visited: the fostering of a sense of community and belonging. We found that the managers, support workers, and relatives were not simply aiming to create a specific world adapted to what they believed was necessary for people with intellectual disabilities to live ‘good lives’; rather, they sought to craft a ‘community’ in which all people were enabled and encouraged to rely on one another. It was this deliberate crafting of community that granted the institutions the characteristics of a place ordered differently from ‘regular’ places and which made them ‘communities of difference’.

In what follows, we discuss four clusters of goods dominant in sheltered living institutions to give a sense of how community is purposefully crafted and how these heterotopic qualities are effectively established. These clusters of goods—namely, ‘shelter’, ‘rhythm’, ‘encounters’, and ‘contribution’—were all geared towards promoting interdependent relationships through shared living and thereby bringing a community into being.

#### Shelter

The first cluster of goods brought into practice at the living institutions is summarised here as ‘shelter’. We place goods such as *protection, isolation, shelter, safety,* and *demarcation* under the heading of ‘shelter’. All these goods are geared towards shaping a safe place in which people feel sheltered, in both literal and figurative senses.

In the introduction, we explained that institutions in the Netherlands are seen as ‘sheltered living arrangements’ due to their *location* and *demarcation* and because all facilities for daily living are present on the sites. In that sense, one could say that shelter is a given, rather than an outcome. However, we found that most institutions actively seek to maintain and cultivate their isolated and demarcated position in order to create a ‘community of difference’. Regarding the location and demarcation of the sites, we found that boundaries were set by fences or gates, with the boundaries of the terrain demarcated by trees or bushes. A sign introducing the institution explains to visitors that they are entering a specific, separated, and ‘different’ place. In this, we can recognise the description of Foucault’s heterotopia as a place that is ordered differently in *space*, as heterotopias are connected to the rest of space but also set apart from it (Foucault 1986, p. 183). The remote location and demarcation of most institutions makes it possible for the sites to exist in ‘a world of their own’, which – according to our respondents – adds to the feeling of shelter and illustrates the different order of things.

Further contributing to this feeling are the pace, sound, and atmosphere of the institution, all of which are adapted to the needs of the people living there. In terms of *pace*, it is notable that time is ordered differently to the manner in ‘regular’ Dutch society, as in the institution, it does not appear to play a strict role in daily life at all. The pace of everyday life is very relaxed, and support workers and residents use time more as a guideline than a deadline. The *sound* of the institutions is also notable. Most of the sites are remarkably quiet, with hardly any loud noises. Since all facilities needed for daily living are available on site, there is no need for cars or buses – which means that residents can move around in peace, and relatives need not fear traffic hazards. Regarding the *atmosphere*, it was striking to note how much attention had been paid to material presentation. The buildings and their interiors were not merely functional (a major critique of institutions, expressed by scholars such as Wolfensberger [1972]) but had been designed and attentively decorated. This was especially true at the anthroposophical institutions, where soft warm fabrics and colours contributed to a sense of shelter. In short, the adaptation of pace, sound, layout, and atmosphere reinforced the sense of being in another world and contributed to a feeling of being sheltered. One of the relatives concisely summarises this crafting of shelter:

So, this is just a safe, quiet environment, in which the pace of life is calm and where there’s everything he [her son] needs and wishes. And because he’s here a lot, he is known and, yes, that provides a bit of security and safety for him. (V4, relative)

#### Rhythm

During the fieldwork, we were struck by the degree of repetition that we found in the daily practices of the sheltered living institutions. We found such repetition in the numerous rituals, feasts, and celebrations that marked the passing of time at these sites. By pursuing the goods of *predictability, structure, familiarity,* and *clarity* in these practices, the managers and support workers promoted individual and collective rhythms for the people living in the institutions. This rhythm provided structure and gave people direction during the days, weeks, and years. Moreover, the rituals provided a common rhythm that guided the people living in the institutions throughout their lives. They gave direction and substance, as well as a sense of collective identity. A professional explains the importance of the shared rhythm:

And beyond that, all the festivities and celebrations are important. Actually, we move from celebration to celebration, from theme to theme (…) It gives direction. Then you know where you are in the year and what is still to come; and yes, that is very nice. I have also worked in another care institution and, there, everyday just looked the same … [*sigh*] … Maybe it was special when we celebrated St Nicholas, but that was about it. (P1, professional)

A manager explains how the collective rhythm contributes to crafting a community:

It [the collective rhythm] also strengthens their bonds with each other and with nature. So, there is rhythm and structure and it strengthens our community. (B2, manager)

As explained, the rhythm is given shape by celebrations of moments and festivities. At the anthroposophical institutions, St Michael, St John, and other days named after saints are celebrated. In addition to these feasts, other moments are also celebrated, such as a bi-weekly Friday evening theatre performance, monthly coffee mornings, and so on. The exact festivities were different for each organization, but what all the sheltered living institutions had in common was a set of traditions that marked the passing of time.

Various rituals accompany the feasts and celebrations. At St John’s, for example, residents of one institution collectively jump over a small fire to mark a new beginning. At another institution, the feast of St Maarten is marked with crafting lanterns. Rituals are not only notable during parties and celebrations, they also mark moments during the day. For example, at the beginning or the end of an activity, songs are frequently sung to accompany daily transitions that, our informants stressed, can be difficult for some residents. At some institutions, the day begins and concludes with gatherings in which managers, support workers, and residents all come together for about 20 min to sing, recite proverbs, and greet one another by forming a circle and holding hands.

Many of the rituals aim to provide a sense of affirmation—showing that participants are recognised, acknowledged, and part of the community:

They sing the song that was also sung on Monday, during my presence at the day opening. When this song is over, they sing another kind of welcome song, in which they also mention the names of everyone present—‘Good morning, Robert. Good morning, Femmianne’, and so on. (PO S3, December 2019)

The feasts, celebrations, and associated rituals provide a rhythm that is experienced by the whole community. Together, the residents live from one ceremony to the next, from one feast to the next, and from one celebration to the next. In this way, the rhythm of the institution seeks to establish a sense of shared direction and a collective ‘different’ identity.

#### Encounters

Another cluster of ‘goods’ we distinguish is ‘encounters’. This set of goods includes *relationality, interaction, contact,* and *being involved* and reveals a normative orientation towards the importance of the social aspects of being human. Encounters are actively encouraged by the sheltered living institutions because they recognise that people are social beings who need one another for their own development. One of the relatives talks about this vision:

The core values of what happens here are related to the vision of what makes someone a human being. That is a belief that people are social beings and that people flourish when they can interact with others. (V3, relative)

One of the managers talks about the value of encounters:

I: What is important for a good life for people with intellectual disabilities?

R: Yes, for me, it is really that you are seen for who you are, that on that basis you can and may enter into contact with other people. You need other people for your own development. So, it’s important that we facilitate—as much as possible—people having contact with others. (B5, manager)

At the sheltered living institutions, people inevitably see one another all the time. People meet one another during the morning ‘opening’ and the day ‘closing’ ceremonies; during the daily, weekly, and monthly festivities and celebrations; and, of course, in the houses and workplaces and at the joint hobby clubs. Moreover, the demarcation of the sites means that people know exactly who belongs to the community and who does not. As researchers, we were frequently noticed as guests and asked, ‘Hey, are you new here?’, and ‘Are you here for an internship?’ Similarly, the residents also notice each other. They stop to greet one another and, when possible, they have conversations. One relative explains why she values her son and herself being ‘noticed’ in this way:

People are socially included on the sites. Yes, and Eric [*who has a severe intellectual disability*] doesn’t notice that, but there are all these people who know him. Then I walk here and they are saying, ‘Oh hi Eric!’, ‘Oh have a good day, Eric’s mother’, you know? (V1, relative)

Because community is seen as very important, support workers encourage interactions in a variety of ways. Residents who naturally get along well with each other, but who live in different homes, are paired as friends, visiting each other regularly during the week and on their respective birthdays. (Short distances between homes also ease the process of maintaining a friendship.) Additionally, the support workers promote interaction during normal daily routines. At one group home for people with profound intellectual and multiple disabilities, the support workers encouraged interaction between residents:

Professional Kees jokes with Anna (who is not able to speak), about resident Gert who sits at the other table and who isn’t able to speak either. Professional Kees states, teasingly, ‘Gert should do the dishes in a minute because he really doesn’t do anything, don’t you think Anna?’ Anna laughs exuberantly. She moves her head side to side in a rhythmic motion, while making high moaning sounds. Gert also laughs. (PO S1, June 2019)

Because the sheltered living institutions are demarcated and isolated and interactions are encouraged, people typically know each other. This gives the residents a sense of security, as they know the people on the site and there are few unexpected visitors. Our informants emphasised that ‘knowing’ each other means that people do not need to pretend to be more competent than they are: they can just be themselves and difference is accepted. A woman who worked in the clothing store in one of the sheltered living institutions confirmed the value of being in a place in which people know each other and in which difference is accepted:

Nobody is surprised if someone wants to lie down on the floor for a while to get their pants on. We’re used to deviant bodies. We’re not surprised by anything. (PO S1, June 2019)

Interestingly, in line with the observations of McKearney (2017) in L’Arche communities, we observed that support workers did not simply seek to encourage residents to master independence (as is typical for support work ‘in the community’); they actively encouraged them to get to know each other and to rely on one another. There was a firm belief that people can flourish in dependent relationships. We also saw this belief reflected in the final cluster of goods, which we will now describe.

#### Contribution

The last cluster of goods to discuss concerns the value of *contribution*. In the daily efforts of the managers and support workers, we observed a normative inclination to encourage people with intellectual disabilities to actively discover their own qualities and identify how they could put these into practice in and for the community. These goods included *participation, giving, doing things yourself,* and *being useful*. One of the directors summarises the core of ‘contribution’ as follows:

Everyone matters. Some of the residents can fulfil a small role, others can fulfil a larger role, but everyone in this community matters. Even if you can only hold a small bucket of clothespins, you are actually helping to hang the laundry and that is of importance too, because if you do the laundry, then we all have clean clothes. […] I think it is very important to realise that everyone has something to offer in this community. (B1, manager)

A community is cultivated through daily practices in which all people are encouraged to participate. During day care, for example, residents perform the tasks necessary for community living. One resident fills bottles with shampoo and another with laundry soap. Other residents take these bottles to the store at which the residents do their shopping and which is staffed by residents with intellectual disabilities. All these little tasks are precisely coordinated so that the communities run like well-oiled machines. The support workers encourage people to rely on one another and depend on one another (instead of mastering independence), thereby stimulating the development of a caring community. One of the support workers explains this process:

In this group home, we encourage people to make use of each other’s qualities. So, when one resident doesn’t have the capacity to pour out tea, but another can, then the other resident can do it. We try to be open about the potential of all the group members and we encourage them to make use of each other and to help each other. (P15, professional)

The support workers also encourage people with severe intellectual disabilities and more challenging behaviour to contribute. We observed that the support workers actively sought tasks for this purpose:

At our sheltered living institution, we house a large group of boys and men with challenging behaviour. For this group, we actively seek out tasks that fit their needs and capacities. Now, they pick up the dirty laundry and bring back the clean laundry, and they deliver the groceries to the various group homes on the site. So, they do matter a lot to our community. (P16, professional)

In short, the person’s ‘difference’ is taken into account, and consideration is given to how this person can contribute to the community. By making contributions, a person is recognised as a member of a community, while their particular needs are also acknowledged. One of the relatives concisely summarises this ideal:

Social inclusion as a right for every person: that is very important. But then, they also have to identify the needs of the specific people. They shouldn’t decide only from the point of view of wider society what social inclusion should look like. No, they have to sort out what social inclusion means for this specific person, and for him, and what does it mean for her. And actually, social inclusion doesn’t necessarily mean that they need to live in a normal house in a normal neighbourhood and be allowed to stand in traffic every day on their way to the day-care activity centre. No, social inclusion means just ‘being part of the whole’ here. (V6)

## Discussion

In this article, we have developed a picture of how the ‘good life’ for people with intellectual disabilities is imagined by managers, support staff, and relatives—identifying the specific shapes that this vision takes in everyday life in three sheltered living institutions in the Netherlands. We painted this ethnographic picture to explore what these places might show us about how difference is perceived and dealt with in Dutch society and about the social inclusion of people with intellectual disabilities and the underlying sameness-difference debate. We reflect on these questions in what follows. Again, in line with the empirical ethics of care approach, we do not seek to pass judgement on the desirability of sheltered living institutions ourselves. The aim is rather to explore the normative repercussions of organizing care through alternative notions of the good life for people with intellectual disabilities.

The three sheltered living institutions we discussed here seem to provide a radically different take on the nature of a ‘good life’ for people with intellectual disabilities. Ultimately, much of what happens in the sheltered living institutions we studied is geared towards the crafting of ‘a community of difference’, a community catered to the specific needs and capacities of the residents. Viewed in this way, the sheltered living institutions essentially function as what Foucault (1986) calls a heterotopia: a ‘counter-site’ that stands in a critical relationship to mainstream society and in which the ‘regular’ order of things is challenged. This is also how our informants think of their institutions themselves. If, as our informants claim, institutions should be regarded as heterotopias, what does the critical mirror they hold up show us?

On the one hand, the sheltered living institutions we studied can be regarded as ‘counter-sites’ that foreground difference, in a critique of Dutch mainstream society. Managers, support staff, and relatives craft ‘communities of difference’ because they believe that the characteristics of ‘normal’ Dutch society—such as its emphasis on autonomy, independence, intellectual capacity, and other liberal values—make wider society unfit for people with intellectual disabilities, whom they believe cannot live up to these norms and are therefore excluded from society. Managers, support staff, and relatives state that Dutch society ‘disables’ people who ‘differ’. Therefore, in their eyes, social inclusion means belonging to a community of peers, specifically crafted for and according to the needs of people with intellectual disabilities. In these sheltered living institutions, goods and values—such as ‘shelter’, ‘rhythm’, ‘encounter’, and ‘contribution’—are given shape, underlining that people are dependent beings who need each other for a good life.

In addition to providing a critique of Dutch mainstream society, the three sheltered living institutions can be regarded as questioning the dominant rhetoric of normalisation and its emphasis on sameness as the core value underpinning social inclusion. The sheltered living institutions challenge us to reconsider the merit of organizing care in the same strict way for all people with intellectual disabilities, regardless of their individual needs and preferences. In the eyes of our respondents, social inclusion happens elsewhere, in a separate ‘community of difference’—challenging the idea of a single ‘public’ or ‘social’ space in which individuals either find themselves included or do not (Simplican and Leader 2015).

On the other hand, the three institutions show us a ‘different’ place—removed from society, at best, or segregated from it, at worst. As a result of the institution’s isolated and segregated position, people without intellectual disabilities cannot meet the residents of these institutions to encounter their different needs and capacities. The three institutions could therefore be accused of *sustaining* ‘normalcy’. Such an accusation would stress that the choice of institutional life leaves the norms and values in mainstream society unquestioned, far from encouraging their adaptation to the needs of people with intellectual disabilities. As a result, people without disabilities continue to set the standards according to which all people are to be measured—standards that can render people with intellectual disabilities stigmatized as ‘deviant’. The accusers would argue that special communities for people with intellectual disabilities allow wider structural and attitudinal factors related to how the residents are perceived, to remain unquestioned. Viewed in this way, the critique of mainstream society posed by sheltered livings institutions fails to make itself known and effectively remains toothless – perhaps leading only to further marginalization. This relinquishing of the principle of ‘sameness’ thus ultimately results in social *exclusion*.

How can we position these three sheltered living institutions in the debate about sameness-difference as a way of promoting social inclusion? Interestingly, the three sheltered living institutions we studied do not seek to exert pressure in the political or public debates about the social place of people with intellectual disabilities, nor do they embrace the principle of difference as a way of promoting social inclusion in ‘mainstream’ society. Rather, they craft a ‘community of difference’ as an end in itself. In this, they differ entirely from the places described by Hall (2004, 2010) or McKearney (2017), which, according to these authors, emphasise difference as a way to strengthen self-identity and to promote equality and social inclusion. They also differ entirely from the three other sheltered living institutions that were part of our larger study, which were engaged in a difficult balancing act between ‘sameness’ and ‘difference’ (Bredewold and Van der Weele forthcoming). The three sheltered living institutions we discussed in this article seem uninterested in negotiating or compromising between the principles of sameness and difference—which is what Young (1990) advocates and what some scholars in the field of intellectual disability studies have since attempted to do (Arneil 2009; Bredewold et al. 2019; Bredewold 2021). Such theorists have high expectations of society’s ability to accommodate the difference of people with intellectual disabilities and to strike a balance between sameness and difference and enable shared identification between people with and without disabilities in mainstream society. By contrast, one might say that the institutions we studied craft their heterotopias as what Johnson (2006, p. 86) calls ‘an escape route from power’, from the dominant discourse of emphasising sameness and concomitant ideals and values. But—as stated earlier—this withdrawal also entails ceasing any attempt to transform the social spaces that excluded people with intellectual disabilities in the first place.

Moreover, the way these institutions cope with difference seems to indicate what Young (1990) calls an ‘essentialist meaning of difference’ (p. 157), where groups are defined as having different essential natures that are more or less fixed. The institutions do not seem to acknowledge that most people have multiple identifications and that people will be similar in some respects and different in others; rather, they seem to suggest that disability—as some disability theorists have put it—‘is a difference that changes everything’ (Herzog 2018, p. 81). Disability ceases to be one difference among many (e.g., race, class, gender), and instead becomes something ‘that creates an exclusion that is not like others’ (Kristeva in Herzog 2018, p. 81). In this way, it appears that the objective of these institutions is to maximize the difference that comes with disability, rather than striking a balance between ‘sameness’ and ‘difference.’

Unsurprisingly then, what the heterotopic mirror shows probably depends on who is looking into it. Yet regardless of how much their outlooks may differ, all who look in the mirror are at least compelled to ask themselves some uncomfortable questions. Is life ‘in the community’ always best for people with intellectual disabilities? How do Western societies deal with the difference that comes with an intellectual or cognitive disability? To what extent can people with intellectual disabilities choose to live with their peers? Are sheltered living institutions always dehumanising and unpleasant? Why is the question of a ‘good life’ for people with intellectual disabilities seemingly so settled, when it so often remains an open question for those who care for people with intellectual disabilities? These questions are not posed very often, in no small part because they are muzzled by the dominant rhetoric of normalisation (Bjorne 2020; Gleeson 2010; Ingham and Atkinson 2013; Novella 2008). We believe these questions are worth asking, though, as they force us to think critically about the ideals guiding the lives of people with intellectual disabilities—ideals which tend to be dreamt up by the nondisabled majority who support them (Stiker 1999). But knowing how much controversy institutions garner, how much resistance they face, and how much their reputation has been tarnished in years past—who will dare to look into the mirror?

## Footnotes

1 Intellectual disability is both a clinical and a cultural concept. The Dutch Ministry of Health defines intellectual disability as ‘a congenital or developmental disorder in intellectual functioning’, involving ‘disabilities in social functioning and self-reliance’ (‘Verstandelijke beperking: cijfers en context’ 2021). In the Netherlands, intellectual disability is clinically diagnosed by means of an IQ test, the score of which can indicate ‘mild’, ‘moderate’, ‘severe’, or ‘profound’ intellectual disability. Needless to say, the clinical approach to intellectual disability is hotly contested (Carlson 2009), with many authors stressing the decidedly *social* nature of disability as a cultural product of ‘othering’ humans who differ from what is considered ‘normal’. In this article, we approach intellectual disability as a category that shapes our social reality, without seeking to scrutinize the category as such.

2 The concept of normalisation can also be found in the work of Michel Foucault (2003), who uses it to refer to a form of power that disciplines bodies by holding them to idealized norms of embodiment and conduct. Foucault’s analysis of normalisation clearly resonates with disability studies critiques of the normalisation movement (e.g. Drinkwater 2015), but exploring them is beyond the scope of this article. Our use of the term here refers exclusively to the concept developed by authors like Nirje and Wolfensberger.

## Declarations

### Research involving human and animals rights

The Medical Research Involving Human Subjects Act (WMO) did not apply to this study and therefore an official approval of this study by the MREC Utrecht is not required under the WMO.

## References

[CR1] Arneil Barbara (2009). Self-image and modern political theory. Political Theory.

[CR2] Beltman Hendrik (2001). Buigen of Barsten? Hoofdstukken uit de geschiedenis van de zorg aan mensen met een verstandelijke handicap in Nederland 1945–2000.

[CR3] Bigby Christine (2008). Known well by no-one: Trends in the informal social networks of middle aged and older people with intellectual disability five year after moving to the community. Journal of Intellectual and Developmental Disability.

[CR4] Bigby Christine, Knox Marie, Beadle-Brown Julie, Clement Tim (2015). ‘We just call them people: Positive regard as a dimension of culture in group homes for people with severe intellectual disability. Journal of Applied Research in Intellectual Disabilities.

[CR5] Bjorne Petra (2020). As if living like others: An idealization of life in group homes for people with intellectual disability. Journal of Intellectual & Developmental Disability.

[CR6] Bos Gustaaf, Kal Doortje (2016). The value of inequality. Cogitatio, Social Inclusion.

[CR7] Bredewold Femmianne (2021). Struggling with sameness and strangeness: (non)-encounters between people with and without intellectual disabilities in two Dutch neighbourhoods. Journal of Intellectual and Developmental Disabilities.

[CR8] Bredewold Femmianne, Hermus Margot, Trappenburg Margo (2018). Living in the community the pros and cons: A systematic literature review of the impact of deinstitutionalization on people with intellectual and psychiatric disabilities. Journal of Social Work.

[CR200] Bredewold, Femmianne, Alke Haarsma, Marja Jager and Evelien Tonkens. 2019. Convivial encounters: conditions for the urban social inclusion of people with intellectual and psychiatric disabilities. *Urban Studies* 57 (10): 2047–2063.

[CR10] Bredewold, Femmianne and Simon van der Weele. Forthcoming. Hoe wordt een goed leven vormgegeven voor mensen met een verstandelijke beperking die wonen op terreinen? En wat betekent dat voor het denken over sociale inclusie?

[CR11] Carlson Licia (2009). The faces of intellectual disability: Philosophical reflections.

[CR12] Ceci Christine, Pols Jeannette, Purkis Mary Ellen, Foth Thomas, Holmes Dave, Hülsken-Giesler Manfred, Kreutzer Suzanne, Remmers Hartmut (2017). Privileging practices: Manifesto for “new nursing studies”. Critical approaches in nursing theory and nursing research.

[CR13] Culham Andrew, Nind Melanie (2003). Deconstructing normalisation: Clearing the way for Inclusion. Journal of Intellectual & Developmental Disability.

[CR14] Drinkwater Chris, Tremain Shelley (2015). Supported living and the production of individuals. Foucault and the Governmentality of Disability.

[CR15] Forrester-Jones Rachel, Carpenter John, Coolen-Schrijner Pauline, Cambridge Paul, Tate Alison, Hallam Angela, Beecham Jennifer, Knapp Martin, Wooff David (2006). Good friends are hard to find? The social networks of people with mental illness 12 years after deinstitutionalisation. Journal of Mental Health.

[CR16] Foucault Michel, Faubion James D (1967). Different spaces. Aesthetics, method and epistemology: essential works of foucault.

[CR17] Foucault Michel (1970). The order of things.

[CR18] Foucault Michel (1986). Of other spaces. Diacritics.

[CR19] Foucault Michel, Marchetti Valerio, Salomoni Antonella (2003). Abnormal: Lectures at the Collège de France, 1974–1975. Translated by Graham Burchell.

[CR20] Gleeson Brendan (2010). Counterpoints of care: Two moments of struggle. Journal of Intellectual Disability Research.

[CR21] Goffman Ervin (1963). Behavior in Public Places. Notes on the Social Organization of Gatherings.

[CR22] Hall Edward (2004). Social geographies of learning disability: Narratives of exclusion and inclusion. Area.

[CR23] Hall Edward (2010). Spaces of social inclusion and belonging for people with intellectual disabilities. Journal of Intellectual Disability Research.

[CR24] Herzog Dagmar (2018). Unlearning Eugenics. Sexuality, Reproduction and Disability in Post-Nazi Europe.

[CR25] Hetherington Kevin (1997). The Badlands of Modernity: Heterotopia and Social Ordering.

[CR26] Ingham Nigel, Atkinson Dorothy (2013). An oral history of the ethics of institutional closure. Ethics and Social Welfare.

[CR27] Johnson Peter (2006). Unravelling foucault’s different spaces. History of the Human Sciences.

[CR28] Johnson Peter (2013). The geographies of heterotopia. Geography Compass.

[CR29] Johnson, Peter. 2016. *Interpretations of Heterotopia (revised)*. Heterotopian Studies. http://www.heterotopiastudies.com

[CR30] Lambek Michael, Das Veena, Fassin Didier, Webb Keane (2015). Four lectures on ethics, anthropological perspectives.

[CR31] Mans Inge (2016). Het hart van de zorg. Idealen en praktijken in de verstandelijk gehandicaptenzorg bij de Hafakker (1960–2010).

[CR32] Mansell Jim (2006). Deinstitutionalisation and community living: Progress, problems and priorities. Journal of Intellectual and Developmental Disability.

[CR33] Mattingly Cheryl, Throop Jason (2018). Anthropology of ethics and morality. Annual Review Anthropology.

[CR34] McDonald Seonaidh (2005). Studying actions in context: A qualitative shadowing method for organizational research. Qualitative Research.

[CR35] McKearney Patrick (2017). L’Arche, Learning disability and domestic citizenship: Dependent political belonging in a contemporary british city. City & Society.

[CR36] Meininger Herman (2010). Connecting Stories: A narrative approach of social inclusion of persons with intellectual disability. Alter.

[CR37] Nirje Bengt (1985). The basis and logic of the normalization principle. Australian and NewZealand Journal of Developmental Disabilities.

[CR38] Novella Enric (2008). Theoretical accounts on deinstitutionalization and the reform of mental health services: A critical review. Medicine, Health Care and Philosophy.

[CR39] Oliver Michael (1993). The politics of disablement.

[CR40] Pols Jeannette (2005). Enacting appreciations: Beyond the clients perspective. Health Care Analysis.

[CR41] Pols Jeannette (2015). Towards an empirical ethics in care: Relations with technologies in health care. Medicine Health Care and Philosophy.

[CR42] Quinlan Elizabeth (2008). Conspicuous invisibility: Shadowing as a data collection strategy. Qualitative Inquiry.

[CR43] Reerink Antoinnette, The Anne-Mei, Roelofsen Eline (2017). Van burger-client naar perspectief van waardigheid. NTZ.

[CR44] Silvers Anita (1995). Reconciling equality to difference: Caring (f)or justice for people with disabilities. Hypatia.

[CR45] Simplican Stacy, Leader Geraldine (2015). Counting inclusion with Chantal Mouffe: A radical approach to intellectual disability research. Disability & Society.

[CR46] Simplican Stacy, Leader Geraldine, Kosciulek John, Leahy Michael (2015). Defining social inclusion of people with intellectual disability and developmental disability: An ecological model of social networks and community participation. Research in Developmental Disabilities.

[CR47] Stiker Henri-Jacques (1999). A history of disability.

[CR48] Taylor Steven J, Bogdan Robert (1989). On accepting relationships between people with mental retardation and non-disabled people: Towards an understanding of acceptance. Disability, Handicap & Society.

[CR50] Van der Weele, Simon. 2022. The moral charge of dependency. Contestations of dependency in care theory and care practice. Doctoral thesis. University of Humanistic Studies Utrecht.

[CR51] van der Weele Simon, Bredewold Femmianne (2021). Shadowing as a qualitative research method for intellectual disability research: Opportunities and challenges. Journal of Intellectual & Developmental Disability.

[CR52] van der Weele Simon, Bredewold Femmianne, Leget Carlo, Tonkens Evelien (2021). The group home as moral laboratory: Tracing the ethic of autonomy in Dutch intellectual disability care. Medicine, Health Care & Philosophy.

[CR53] ‘Verstandelijke beperking: cijfers en context.’ 2021. Volksgezondheidenzorg.info.

[CR54] Wiesel Ilan, Bigby Christine (2015). Movement on shifting sands: Deinstitutionalisation and people with intellectual disability in Australia, 1974–2014. Urban Policy and Research.

[CR55] Wolfensberger Wolf (1972). The principle of normalization in human services.

[CR56] Wolfensberger Wolf (1983). Social Role Valorization: A proposed new term for the principle of normalization. Mental Retardation.

[CR57] Young Iris Marion (1990). Justice and the politics of difference.

